# LncRNA-Fendrr protects against the ubiquitination and degradation of NLRC4 protein through HERC2 to regulate the pyroptosis of microglia

**DOI:** 10.1186/s10020-021-00299-y

**Published:** 2021-04-15

**Authors:** Li-Qing Wang, Yue-Ying Zheng, Heng-Jun Zhou, Xiong-Xin Zhang, Pin Wu, Sheng-Mei Zhu

**Affiliations:** 1 Department of Anesthesiology, The First Affiliated Hospital, College of Medicine, Zhejiang University, 79 Qingchun Road, Hangzhou, 310003 People’s Republic of China; 2 Department of Neurosurgery, The First Affiliated Hospital, College of Medicine, Zhejiang University, Hangzhou, 310003 People’s Republic of China

**Keywords:** Cerebral ischemia–reperfusion, Diabetes, Pyroptosis, LncRNA-Fendrr, NLRC4, HERC2

## Abstract

**Objectives:**

Targeted inhibition of inflammatory response can reduce diabetic cerebral ischemia–reperfusion (I/R) injure. Pyroptosis is characterized by caspase-1 dependence and the release of a large number of pro-inflammatory factors. LncRNA-Fendrr is associated with a variety of diseases, but Fendrr has not been studied in diabetic cerebral I/R. NLR-family CARD-containing protein 4 (NLRC4) regulate the pyroptosis of microglia cells. This study was designed to investigate whether Fendrr is involved in the effects of diabetic cerebral I/R injury.

**Methods:**

The diabetic brain I/R model in mice was constructed. Mouse microglia cell line BV-2 cells were exposed to high glucose followed by hypoxia/reoxygenation (H/R). Fendrr and some pyroptosis-associated proteins were detected by qRT-PCR, western blot or ELISA. HE staining was used to detect pathological changes. Microglia pyroptosis was detected by TUNEL staining. RNA pull-down and RNA Immunoprecipitation were used to detect binding of Fendrr to HERC2 (E3 ubiquitin ligase), and CO-IP detected binding of HERC2 to NLRC4. The ubiquitination of NLRC4 was detected by ubiquitination experiments.

**Results:**

Fendrr was significantly increased in the diabetic cerebral I/R model, and NLRC4 inflammatory complex and pyroptosis mediated inflammatory factors were increased. NLRC4 and inflammatory cytokines associated with pyroptosis were decreased in the high glucose-treated hypoxia/reoxygenation (H/R)-induced microglia after Fendrr knockdown. Fendrr bound to HERC2 protein, and HERC2 bound to NLRC4. Meanwhile, Fendrr could inhibit the ubiquitination of NLRC4, HERC2 promoted the ubiquitination of NLRC4 protein. Moreover, the effect of Fendrr overexpression in the diabetic cerebral I/R model of microglia can be reversed by HERC2 overexpression.

**Conclusion:**

Fendrr can protect against the ubiquitination and degradation of NLRC4 protein through E3 ubiquitin ligase HERC2, thereby accelerating the pyroptosis of microglia.

## Introduction

Cerebral ischemia–reperfusion (I/R) injury is severe brain dysfunction occurs when blood supply returns to tissue after a period of time of brain ischemia (Stegner et al. 2019). Cerebral I/R injury can cause neuronal damage and death, and lead to neurological deficits (Deng et al. 2013). Some studies have shown that diabetes can increase inflammation induced by cerebral I/R, thereby aggravating neurological damage (Feigin 2005). Therefore, it is important to explore a neuroprotective mechanism that may mitigate I/R injury in the diabetic state. Some research data showed that microglia has a specific effect on neuronal injury after ischemic injury (Ma et al. 2017). Excessive activation of microglia cells produces a large number of pro-inflammatory cytokines, resulting in neuronal necrosis and apoptosis, which further aggravates diabetic cerebral I/R injury (Huang et al. 2015). However, the specific mechanism of inflammatory response of microglia after diabetic cerebral I/R injury is still unclear.

Pyroptosis, a newly discovered programmed cell death, is characterized by activation of inflammasome and activation of the cysteinyl aspartate specific proteinase-1 (caspases-1) (Bergsbaken et al. 2009). Inflammasome is the multi-protein complex composed of NLR (NOD-like receptor), ASC (apoptosis-associated speck-like protein containing CARD) and caspase protease (Gross et al. 2011). Inflammasome activates interleukin 1β (IL-1β) and interleukin 18 (IL-18) by activating caspase-1, ultimately affecting pyroptosis (Gross et al. 2011; Abderrazak et al. 2015). NLR-family CARD-containing protein 4 (NLRC4) inflammasome was significantly increased in the ischemia–reperfusion injury model, and the inhibition of NLRC4 expression reduced pyroptosis of microglia cells under ischemic conditions (Poh et al. 2019). These studies suggest that NLRC4 may be a potential target for the treatment of brain transfusion reperfusion injury. HERC2 is an E3 ubiquitin ligase with multiple structural domains, and acts as a scaffold to link protein complexes to affect key cellular pathways (Galligan et al. 2015). It was predicted by software that HERC2 is an E3 ubiquitin ligase of NLRC4. Whether HERC2 participates in cerebral I/R injury regulated by NLRC4 inflammatory body requires further study.

Long non-coding RNA (lncRNA) refers to non-coding RNAs longer than 200 nucleotides. There is growing evidence that lncRNA can regulate gene expression levels at multiple levels, making it play a crucial role in a variety of diseases (Li et al. 2014). For instance, lncRNA small nucleolar RNA host gene 12 (SNHG12) induces autophagy activation to mitigate cerebral I/R injury (Yao et al. 2019). LncRNA FOXF1 adjacent non-coding developmental regulatory RNA (Fendrr) was first discovered in mouse mesoderm and plays an indispensable role in the development of mouse heart and body wall (Grote et al. 2013). LncRNA-Fendrr has been shown to be involved in many diseases, such us gastric cancer, osteosarcoma, cholangiocarcinoma (Xu et al. 2014; Kun-Peng et al. 2017; Qin et al. 2019). Fendrr has been reported to promote the apoptosis of human brain microvascular endothelial cells in hypertensive intracerebral hemorrhage (Dong et al. 2018). However, the role of Fendrr in diabetic cerebral I/R injury has not been reported. This study was to investigate whether Fendrr in microglia is involved in diabetic cerebral I/R injury.

## Materials and methods

### Animals

Male C57BL/6 mice (5–7 weeks old) were purchased from Shanghai Sippr-BK laboratory animal Co. Ltd. All experimental protocols were approved by the Animal Experimentation Ethics Committee of the First Affiliated Hospital of Zhejiang University. Mice were induced to develop type II diabetes by 3 weeks of high-fat diet, followed by intraperitoneal injection of streptozocin (STZ, 20 mg/kg body weight), and then high-fat feeding for another week. Mice in control group were injected intraperitoneally with normal saline on the basis of normal diet. The establishment of a diabetic mouse model was considered successful when the blood glucose concentration is higher than 10 mmol/L in the blood samples collected from the tail vein, which was measured by a blood glucose tester (Blood Glucose Te, USA) (Hong et al. 2018). Eight weeks after injecting STZ (Sigma, MO, USA), the cerebral ischemia/reperfusion (I/R) injury model was constructed through middle cerebral artery occlusion (MCAO) (Zhao et al. 2019). The mice were randomly divided into four groups: normal mice (Control-sham group), normal mice with cerebral I/R injury (Control-I/R group), mice underwent sham operation group (DM-sham group) and diabetic mice with cerebral I/R injury (DM-I/R group), n = 10/group. Three mice in each group were randomly selected for hematoxylin–eosin staining, three mice in each group were subjected to immunofluorescence detection, and the remaining mice in each group were decapitated, and the brain tissue was cryopreserved for subsequent detection.

### Cell culture

Mouse microglia cell line BV-2 cells were purchased from the Cell Resource Center, Shanghai Institutes for Biological Sciences. BV-2 cells were cultured in DMEM (Gibco, New York, USA) supplemented with 10% foetal bovine serum (Biological Industries, Israel) and 1% penicillin/streptomycin 100X (Solarbio, Beijing, China) at 37 °C in 5% CO_2_.

In the high glucose control group (HG-control), cells were treated for 48 h under high glucose (33 mM) conditions. In the high glucose hypoxia/reoxygenation group (HG-H/R), after treated for 48 h under high glucoseconditions, cells were subjected to hypoxia/reoxygenation (H/R) treatment. The cells were cultured in the 95% N_2_ and 5% CO_2_ hypoxia environment for 3 h, and then reoxygenated in fresh medium for 3 h (Hu et al. 2016).

### Cell transfection

The si-NC, si-Fendrr and si-HERC2 are synthesized by ribobio (Guangzhou, China). pcDNA3.1-Fendrr, pcDNA3.1-HERC2 and empty vector pcDNA3.1 were synthesized by Invitrogen (Carlsbad, CA, USA). According to the instructions, Lipofectamine 2000 (Invitrogen) was used to transfect the cells. Cells were collected after 24 h.

### Quantitative real-time PCR (qRT-PCR)

Total RNA was extracted from mouse brain tissues or BV-2 cells using the RNAsimple Total RNA Extraction Kit (Tiangen, Beijing, China). RNA was reverse-transcribed using the InRcute lncRNA cDNA Synthesis Kit (Tiangen). QRT-PCR was performed on ABI 7500 Real-Time PCR system (Applied Biosystems, Carlsbad, USA) using SYBR Premix Ex Taq (TaKaRa). The relative expressions of lncRNA were normalized to β-actin, and calculated by the 2^−ΔΔCt^ method. Sequences of the primers are shown below: Fendrr, 5′-CTGCC CGTGT GGTTA TAATG-3′ (forward) and 5′-TGACT CTCAAG TGGGT GCTG-3′ (reverse); the control β-actin, 5′-CATGT TTGAG ACCTT CAACA CCCC-3′ (forward) and 5′-GCCAT CTCCT GCTCG AAGTC TAG-3′ (reverse).

### Western blotting (WB) and co-immunoprecipitation (Co-IP)

Cells or tissues were lysed using RIPA lysis buffer (Beyotime, Shanghai, China) containing protease inhibitors. The protein concentration was determined according to the instructions of the BCA kit (Beyotime). The protein was denatured at 100 °C for 5 min, subjected to SDS-PAGE, and electro-transferred onto polyvinylidene fluoride (PVDF) membranes (Millipore, Bedford, USA). The membrane was incubated in blocking buffer for 2 h at 4 °C, and then incubated overnight with the corresponding primary antibody. Then, it was incubated with the secondary antibody for 2 h at 4 °C. The band was detected using the BeyoECL (Beyotime). The levels of protein expression were quantified by densitometry and normalized to β-actin expression.

The Co-IP assay was performed on HERC2 and NLRC4 proteins. The supernatant of cell lysates was extracted in accordance with the above steps. Anti-HERC2 antibody (ab85832, Abcam, UK) and protein A agarose beads (Univ-bio, Shanghai) were added and incubated overnight at 4 °C. The agarose beads were centrifuged to the bottom of the tube, and the supernatant was carefully aspirated. Then washing 3 times with lysis buffer, and the Co-IP product was eluted by incubation with 2xSDS loading buffer and boiling for 3 min. Western blotting analysis was performed.

### Hematoxylin–eosin staining

The brain tissues of mice in DM-sham group and DM-I/R group were removed after surgery and rinsed with pre-cooled PBS. After fixation with 4% paraformaldehyde (Sigma) for 48 h, routine paraffin embedding and section were performed. Hematoxylin and Eosin Staining Kit (Beyotime, China) was used for hematoxylin–eosin (HE) staining.

### TUNEL staining

Cell Death Detection Kits (Roche, IN, USA) were used to test pyroptosis in the brain tissues and BV-2 cells following the instructions for testing. The nuclei were stained with DAPI. The image was observed and recorded by a fluorescence microscope. Nuclei labeled with DAPI and TUNEL were considered positive. At least five viewing fields were quantified to obtain each data.

### Immunofluorescence staining

Brain tissue sections were washed with 0.01 M PBST for 5 min and repeated twice. The sections were then placed in 10% BSA wet box (37 °C) for 30 min. Primary antibodies were added and incubated overnight at 4 °C, then secondary fluorescent antibodies were added and incubated for 1 h at room temperature in wet box and 5 times with 0.01 M PBST in the dark, repeated 2 times. Finally, the image was observed under a fluorescence microscope.

### Enzyme linked immunosorbent assay (ELISA)

The levels of IL-1β and IL-18 in cell supernatant or brain tissue homogenate were detected by ELISA kit (eBioscience, California, USA) according to the manufacturer’s instructions.

### RNA immunoprecipitation (RIP)

BV-2 cells were lysed with RIPA lysis buffer and centrifuged. RIP assay using the Magna RIP RNA-Binding Protein Immunoprecipitation Kit (Millipore, USA) and anti-HERC2 antibody (Abcam) or normal mouse immunoglobulin G (IgG; Millipore). The precipitated product was eluted according to the instructions. The expression of Fendrr was detected by qRT-PCR.

### RNA pull-down

Biotinylated lncRNA-Fendrr was purified and then transfected into BV-2 cells. Cells were harvested after 48 h. RNA pull-down test was performed with Pierce Magnetic RNA–Protein Pull-Down Kit (Thermo Fisher Scientific, MA, USA). The lysate of BV-2 cells was incubated with 50 μL of streptavidin agarose magnetic beads (Thermo Fisher Scientific). The RNA–protein complex was purified and tested by Western blotting.

### Ubiquitination assay

Fendrr or Fendrr and HERC2 were overexpressed in BV-2 cells and treated with MG132 (10 μg/mL). The cells were co-transfected with a plasmid expressing HA-tagged ubiquitin (HA-Ub) and a plasmid expressing FLAG-tagged HERC2 (FLAG- HERC2). The cells were directly boiled in 0.2 mL SDS lysis buffer and sonicated to dissolve. The cell lysate was incubated with 5 μg of anti-HA antibody overnight at 4 °C to recover ubiquitinated protein, and detected by Western blotting.

### Statistical analysis

Statistical analyses were performed via SPSS 22.0 statistical software (IBM, Armonk, NY, USA). Measurement data were expressed as mean ± standard deviation (SD). Differences between two groups were assessed using paired t test. If the *P* value was < 0.05, it was considered to be statistically significant.

## Result

### LncRNA-Fendrr and NLRC4-mediated inflammation is activated in the brain tissue of diabetic cerebral ischemia/reperfusion injury mice

**Fig. 1:**
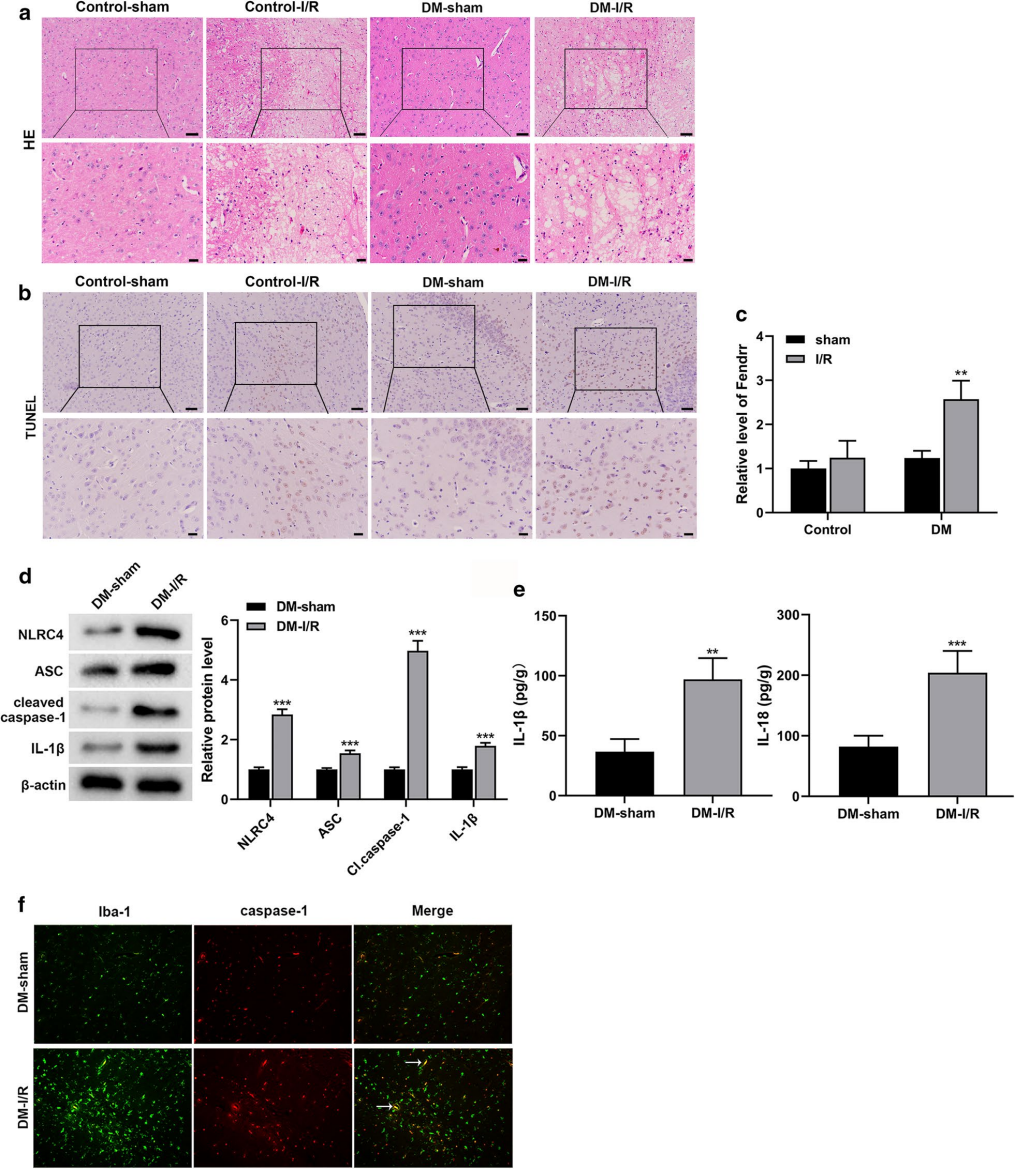
LncRNA-Fendrr and NLRC4-mediated inflammation is activated in the brain tissues of diabetic cerebral ischemia/reperfusion injury mice. The model of cerebral ischemia/reperfusion (I/R) injury in mice was established, and mice were divided into the DM-I/R group and the DM-sham group (control), n = 10/group. **a** HE staining was used to detect pathological changes in mouse brain tissue. Scale bar in the top line is 50 μm, and the bottom line is 20 μm. **b** TUNEL staining was used to detect neuronal apoptosis in mouse brain tissue. Scale bar in the top line is 50 μm, and the bottom line is 20 μm. **c** The expression of lncRNA-Fendrr was detected by qRT-PCR. **d** NLRC4, ASC, cleaved caspase-1 and IL-1β protein levels were detected by Western blotting. **e** The levels of IL-1β and IL-18 in brain tissue homogenate were detected by ELISA. **f** Detecting microglia markers Iba-1 and caspase-1 in the brain tissue of mice by Immunofluorescence (n = 3/group). Data are presented as means ± SD. *P* values were analyzed by Student’s t-test. **P < 0.01 and ***P < 0.001 versus the DM-sham group

Eight weeks after the construction of the mouse diabetes model by injection of STZ, the cerebral ischemia/reperfusion (I/R) injury model was constructed by MCAO (Zhao et al. 2019). No pathological changes were observed in the brain tissues of Control-sham and DM-sham groups. In the Control-I/R group, HE staining revealed that intercellular substance was loose and meshed, the neurons are swollen and the cytoplasm is vacuolated, while this effect in the DM-I/R group was more severe (Fig. 1a). In addition, TUNEL staining showed that the neurons in the control-sham group were normal (Fig. 1b). The number of positive cells in the brain tissues of the DM-Sham group was less, while the number of positive cells was significantly increased in Control-I/R group, and the highest in DM-I/R group (Fig. 1b). The lncRNA-Fendrr expression in the DM-I/R group was obviously higher than that in the Control-sham group, Control-I/R group and DM-sham group (Fig. 1c). In order to test whether NLRC4 inflammasome-mediated inflammatory response occurs, we have tested the relevant factors. The results showed that NLRC4, ASC, cleaved caspase-1 and IL-1β proteins were significantly elevated in the DM-I/R group compared with the DM-sham group (Fig. 1d). The IL-1β and IL-18 levels in the brain tissue homogenate of the DM-I/R group were significantly increased compared to the DM-sham group (Fig. 1e). Detection of microglia markers Iba1 and caspase 1 in mouse brain tissue by immunofluorescence, the results showed that Iba1 and caspase 1 expressions were observably increased, and colocalization signals were also increased in the DM-I/R group compared with the DM-sham group (Fig. 1f).

### Interfering lncRNA-Fendrr inhibits NLRC4-mediated pyroptosis of the high glucose hypoxia/reoxygenation model cells

**Fig. 2:**
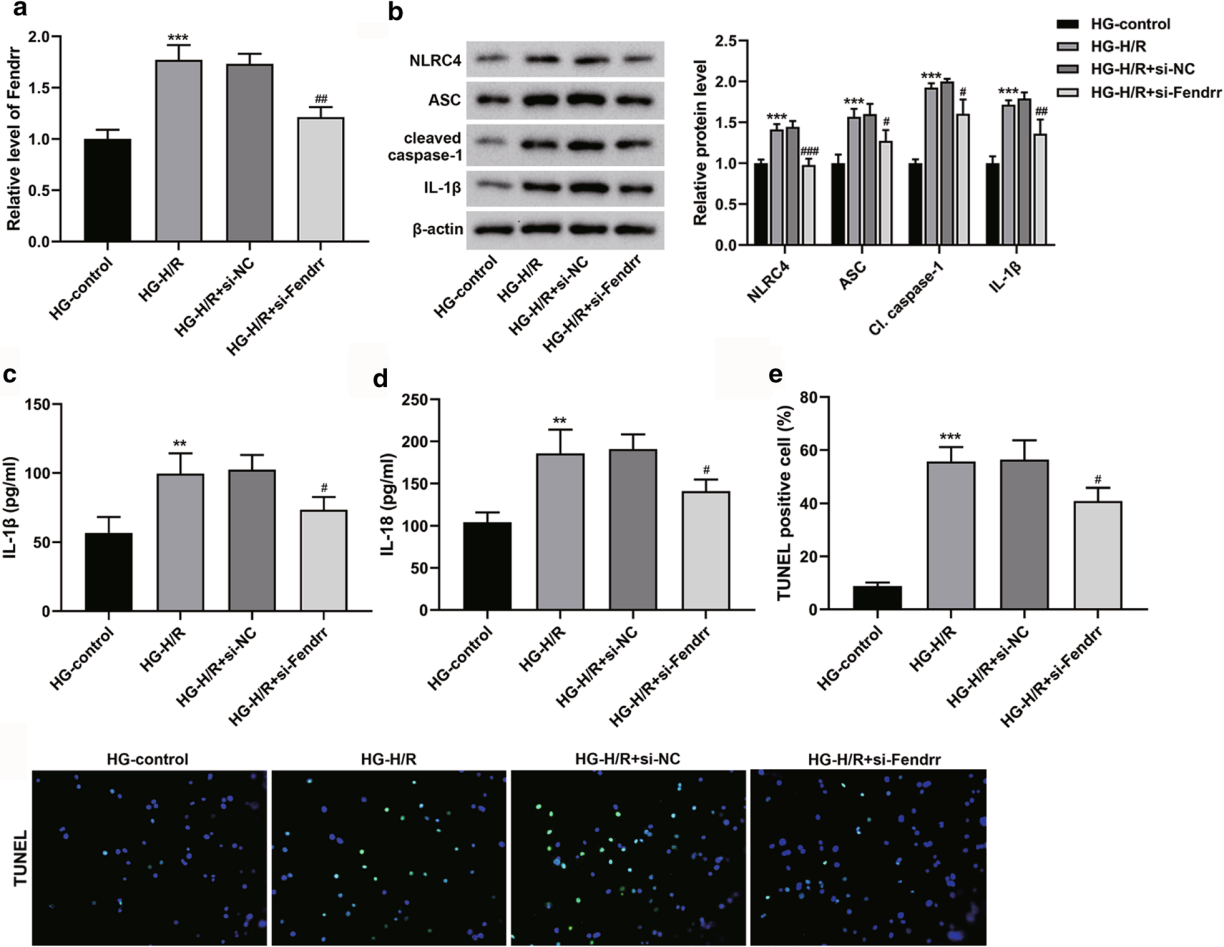
Interfering Fendrr inhibits NLRC4-mediated pyroptosis of the high glucose (HG)-hypoxia/reoxygenation (H/R) model cells. Exposing mouse microglia cell line BV-2 cells to high glucose followed by H/R, and the cells were transfected with si-Fendrr or si-NC (control). The cells were divided into the HG-control group, the HG-H/R group, the HG-H/R + si-NC group, and the HG-H/R + si-Fendrr group (n = 3/group). **a** The expression of lncRNA-Fendrr was detected by qRT-PCR. **b** NLRC4, ASC, cleaved caspase-1 and IL-1β protein levels were detected by Western blotting. **c** and **d** The levels of IL-1β and IL-18 in the cell supernatant were measured by ELISA. **e** TUNEL analysis was performed on the cells. Data are presented as means ± SD. *P* values were analyzed by Student’s t-test. **P < 0.01 and ***P < 0.001 versus the HG-control group; #*P* < 0.05 and ###*P* < 0.001 versus the HG-H/R group

To investigate possible molecular mechanisms, we conducted in vitro studies. We exposed mouse microglia cell line BV-2 cells to high glucose followed by H/R (Hu et al. 2016). These results were consistent with those observed in model mice: the expression of Fendrr was observably elevated (Fig. 2a), and the protein expressions of NLRC4, ASC, cleaved caspase-1 and IL-1β were observably elevated (Fig. 2b), the levels of IL-1β and IL-18 were also obviously elevated in the supernatant (Fig. 2c, d). However, these results were reversed when the lncRNA-Fendrr was knocked down (Fig. 2a–d). Moreover, we performed TUNEL staining, and the results showed that the DNA fracture in the HG-H/R group was elevated compared to the HG-control group. Nevertheless, DNA fracture was lessened after inhibition of lncRNA-Fendrr expression (Fig. 2e). These results suggest that Fendrr can affect the inflammatory-mediated pyroptosis pathway in model cells.

### LncRNA-Fendrr may regulate ubiquitination of NLRC4 protein through HERC2

**Fig. 3:**
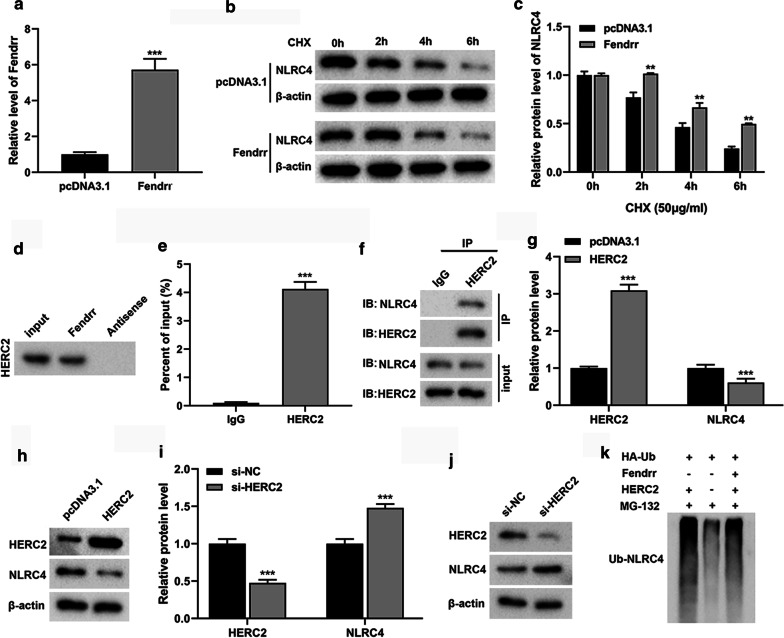
LncRNA-Fendrr may regulate the ubiquitination of NLRC4 protein through HERC2. **a** The expression of Fendrr was detected by qRT-PCR. **b**, **c** After transfection, the cells were treated with Cycloheximide (CHX) (50 μg/mL) for 0, 2, 4 and 6 h, and the NLRC4 protein level was detected through western blot. **d**, **e** The binding of lncRNA Fendrr to HERC2 protein was detected by (**d**) RNA pull-down and (**e**) RNA Immunoprecipitation (***P < 0.001 versus the IgG group). **f** The interaction between HERC2 and NLRC4 was detected by Co-Immunoprecipitation. **g**, **h** pcDNA3.1-HERC2 or pcDNA3.1 (control) was transfected into BV-2 cells, and then HERC2 and NLRC4 were detected by Western blotting. **i**, **j** si-HERC2 or si-NC (control) was transfected into BV-2 cells, and then HERC2 and NLRC4 expressions were detected by Western blotting. **k** BV-2 cells were treated with MG132 (10 μg/mL) and transfected or co-transfected with pcDNA3.1-Fendrr and pcDNA3.1-HERC2 to detect NLRC4 ubiquitination levels. n = 3/group. Data are presented as means ± SD. *P* values were analyzed by Student’s t-test. **P < 0.01 and ***P < 0.001 versus the pcDNA3.1 group

To explore the possible mechanism of lncRNA-Fendrr on NLRC4 regulation, we overexpress lncRNA-Fendrr by transfection in BV-2 cells (Fig. 3a). We treated BV-2 cells with the protein synthesis inhibitor CHX. The results showed that NLRC4 protein was gradually decreased in both groups after 2 h, but NLRC4 protein was higher in the lncRNA-Fendrr overexpression group than the control group at the same time point (Fig. 3b, c). These results indicate that NLRC4 protein is more stable upon lncRNA-Fendrr overexpression.

We found that lncRNA-Fendrr could bind to HERC2 through software prediction (http://www.rna-society.org/raid/index.html). To further verify whether lncRNA-Fendrr binds to HERC2, we performed RNA pull-down and RIP experiments. The results showed that lncRNA-Fendrr can bind to the HERC2 protein (Fig. 3d, e). We predicted the combination of HERC2 and NLRC4 by software (http://ubibrowser.ncpsb.org/). To further verify whether HERC2 binds to NLRC4, we performed Co-IP in BV-2 cells. The results showed that NLRC4 protein was detected in the precipitation of HERC2 (Fig. 3f). Next, we overexpressed HERC2 in BV-2 cells and found that NLRC4 protein was markedly lessened through western blot (Fig. 3g, h). In contrast, NLRC4 protein was obviously elevated after the knockdown of HERC2 (Fig. 3i, j).

To investigate whether lncRNA-Fendrr affects the ubiquitination of NLRC4, we used MG132 to treat cells with lncRNA-Fendrr overexpression for ubiquitination experiments. We found that the extent of NLRC4 ubiquitination was notably reduced in lncRNA-Fendrr overexpressed cells (Fig. 3k). This suggests that lncRNA-Fendrr can protect against the ubiquitination and degradation of NLRC4 protein. However, the overexpression of HERC2 reverses this result (Fig. 3k). So we speculate that lncRNA-Fendrr inhibits the ubiquitination of NLRC4 by suppressing HERC2.

### LncRNA-Fendrr regulates NLRC4-mediated microglial pyroptosis through HERC2

**Fig. 4:**
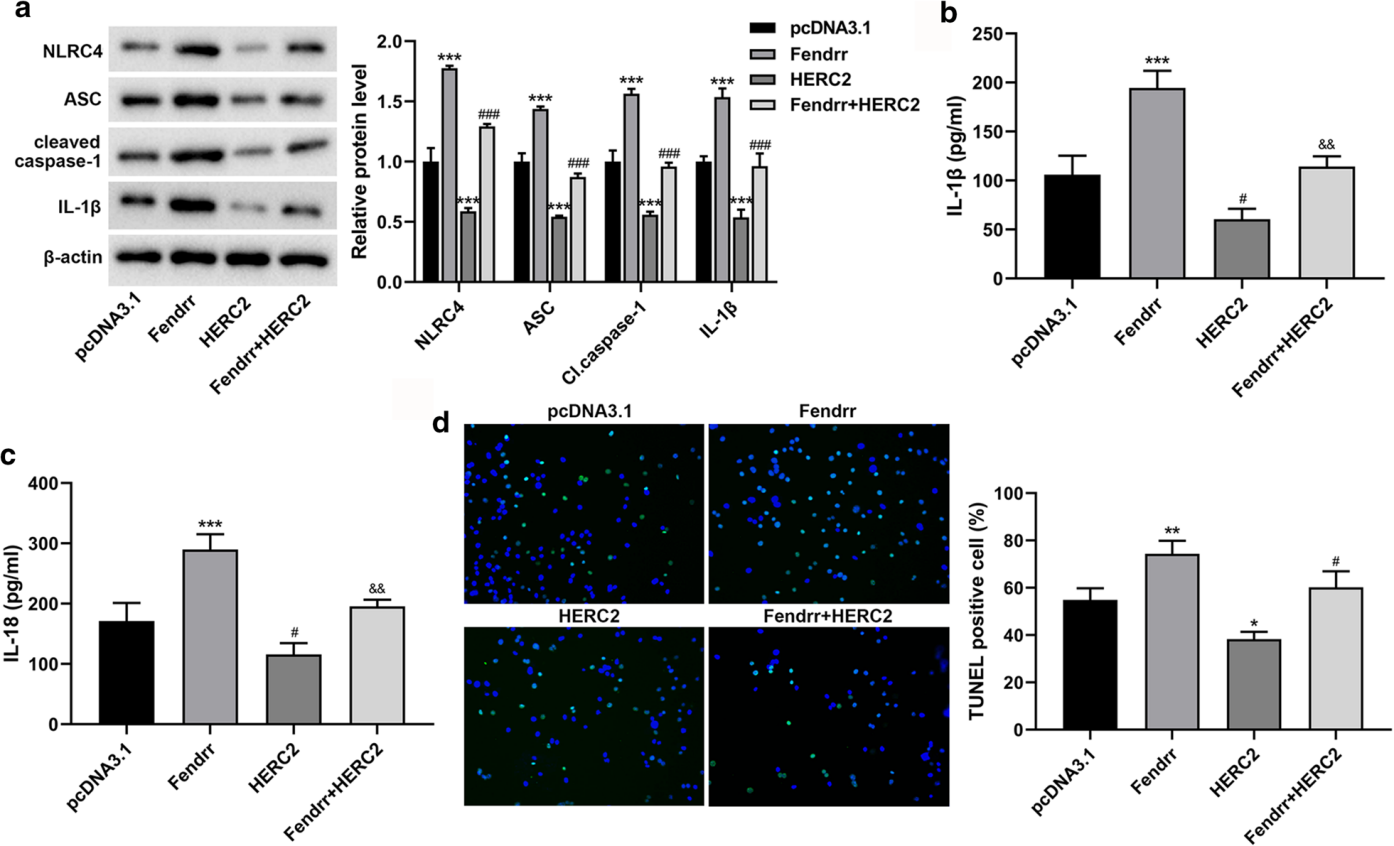
Fendrr regulates NLRC4-mediated microglial pyroptosis through HERC2. Exposing mouse BV-2 cells to high glucose (HG) followed by hypoxia/reoxygenation (H/R), and the cells were transfected or co-transfected with pcDNA3.1 (control), pcDNA3.1-Fendrr and pcDNA3.1-HERC2. The cells were divided into the pcDNA3.1 group, the Fendrr group, the HERC2 group, and the Fendrr + HERC2 group (n = 3/group). **a** NLRC4, ASC, cleaved caspase-1 and IL-1β proteins were detected by Western blotting. **b**, **c** The levels of IL-1β and IL-18 in the cell supernatant were measured by ELISA. **d** TUNEL analysis was performed on the cells. Data are presented as means ± SD. *P* values were analyzed by Student’s t-test. *P < 0.05, **P < 0.01 and ***P < 0.001 versus the pcDNA3.1 group; #*P* < 0.05 and ###*P* < 0.001 versus the Fendrr group; &&*P* < 0.01 versus the Fendrr group

In order to investigate whether lncRNA-Fendrr can regulate NLRC4 mediated microglial pyroptosis through HERC2 in diabetic cerebral I/R injury model in vitro, we transfected or co-transfected pcDNA3.1-Fendrr and pcDNA3.1-HERC2, and exposed BV-2 cells to high glucose followed by H/R. The results showed that after overexpressing lncRNA-Fendrr, the protein expressions of NLRC4, ASC, cleaved caspase-1 and IL-1β were observably elevated (Fig. 4a), the levels of IL-1β and IL-18 were obviously elevated in the supernatant (Fig. 4b, c), and the DNA fracture was also elevated (Fig. 4d). Nevertheless, these results were reversed when HERC2 was overexpressed on this basis (Fig. 4a–d). These results indicate that lncRNA-Fendrr regulates NLRC4-mediated microglial pyroptosis through HERC2 in the model of diabetic cerebral I/R injury.

## Discussion

Cerebral ischemia is one of the most common diseases of the central nervous system, and cerebral I/R may aggravate neuronal death and neurological deficits. Inflammation is considered to be an indispensable step in the progression of cerebral I/R injury, and targeted inhibition of inflammatory response can alleviate cerebral I/R injury (Wu et al. 2017, 2018; Mizuma and Yenari 2017). Diabetes can affect ischemic cerebral I/R injury, compared with non-diabetic patients, cerebral I/R in diabetic patients not only has a higher mortality rate, but also tends to a more severe condition and slower recovery (Sun et al. 2016; Yu et al. 2016). Diabetes can promote inflammatory response caused by cerebral I/R (Feigin 2005). Microglia is a kind of glial cell, which mediates the inflammatory response of ischemic stroke (Yin et al. 2018). Microglia activation activates pro-inflammatory factors to promote neuroinflammation, neuronal necrosis or apoptosis, further aggravating diabetes-cerebral ischemia/reperfusion injury (Huang et al. 2015). Therefore, inhibition of the inflammatory response of microglia is important for diabetes-cerebral I/R injury.

LncRNAs have been shown to be involved in the development and progression of various diseases. Studies have found that lncRNAs are involved in diabetes-cerebral I/R injury. For instance, lncRNA-MALAT1 aggravates diabetes-cerebral I/R injury by triggering an inflammatory response in microglia cells via myeloiddifferentiationfactor88 (MyD88) signaling (Wang and Zhou 2018). Our study found that lncRNA-Fendrr was observably elevated in the mouse model and microglia model of diabetes-cerebral I/R, and the inflammation and cell death pathways were activated. These results suggest that lncRNA-Fendrr may be involved in inflammation and pyroptosis of microglia in diabetes-cerebral I/R.

NLRC4 has been found to be expressed in immune cells (such as microglia) and promotes caspase-1 activation by forming an inflammatory complex with ASC, pro-caspase-1 (Duncan and Canna 2018). Activated caspase-1 converts pro-IL-1β, pro-IL-18 into active IL-1β and IL-18, thereby causing inflammation (Poyet et al. 2001; Zhao and Shao 2015). Pyroptosis is characterized by caspase-1 dependence and the release of a large number of pro-inflammatory factors (Fink et al. 2008; Bergsbaken et al. 1950). Activation of NLRC4 inflammasome plays an important role in the process of pyroptosis (Guo et al. 2018; Cerqueira et al. 1950). Our study found that the restraint of lncRNA-Fendrr markedly reduced NLRC4, ASC, pro-caspase-1, and caspase-1 mediated pyroptosis in BV-2 cell model of diabetes-cerebral I/R. Studies have shown that the restraint of NLRC4 expression under ischemic conditions can inhibit inflammatory factor activity and reduce pyroptosis of microglia (Poh et al. 2019). These results suggest that lncRNA-Fendrr affects the pyroptosis of microglia cells through NLRC4, thereby affecting inflammatory response in diabetes-cerebral I/R.

HERC family is a key component of a variety of cellular functions, including neurodevelopment, cell growth, and immune responses (Mao et al. 2018; Sánchez-Tena et al. 2016). HERC2 is a member of the HERC family, a multi-domain E3 ubiquitin ligase that acts as a scaffold connexin complex to affect key cellular pathways (Galligan et al. 2015). Our study found that lncRNA-Fendrr regulates the ubiquitination of NLRC4 by HERC2. Further studies have shown that lncRNA-Fendrr promotes NLRC4-mediated pyroptosis in microglia model of diabetes-cerebral I/R, but HERC2 reversed the effect of lncRNA-Fendrr. These results indicate that lncRNA-Fendrr regulates microglial pyroptosis, which is partly due to the regulation of ubiquitination of NLRC4 protein through HERC2.

## Conclusion

Our study found that lncRNA-Fendrr is highly expressed in the diabetes-cerebral I/R model and microglia cells treated with high glucose followed by hypoxia/reoxygenation (H/R). Fendrr can protect against the ubiquitination and degradation of NLRC4 protein through E3 ubiquitin ligase HERC2 to increase pyroptosis of microglia. This suggests that Fendrr may be a potential target for the treatment of diabetic cerebral I/R injury.

## Data Availability

Not applicable.

## Abbreviations

I/R: Ischemia–reperfusion
NLRC4: NLR-family CARD-containing protein 4
H/R: Hypoxia/reoxygenation
Caspases-1: Cysteinyl aspartate specific proteinase-1
NOD-like receptor: Multi-protein complex composed of NLR
IL-1β: Interleukin-1β
IL-18: Interleukin-18
ASC: Apoptosis-associated speck-like protein containing CARD
lncRNA: Long non-coding RNA
SNHG12: Small nucleolar RNA host gene 12
STZ: Streptozocin
HG: High glucose

## References

[CR1] Abderrazak A, Syrovets T, Couchie D, El Hadri K, Friguet B, Simmet T, Rouis M (2015). NLRP3 inflammasome: from a danger signal sensor to a regulatory node of oxidative stress and inflammatory diseases. Redox Biol.

[CR2] Bergsbaken T, Fink SL, Cookson BT (2009). Pyroptosis: host cell death and inflammation. Nat Rev Microbiol.

[CR3] Bergsbaken T, Fink SL, den Hartigh AB, Loomis WP, Cookson BT (2011). Coordinated host responses during pyroptosis: caspase-1-dependent lysosome exocytosis and inflammatory cytokine maturation. J Immunol (Baltimore, Md: 1950).

[CR4] Cerqueira DM, Pereira MS, Silva AL, Cunha LD, Zamboni DS (2015). Caspase-1 but not caspase-11 is required for NLRC4-mediated pyroptosis and restriction of infection by flagellated legionella species in mouse macrophages and in vivo. J Immunol (Baltimore, Md: 1950).

[CR5] Deng B, Gou X, Chen H, Li L, Zhong H, Xu H, Jiang F, Zhao Z, Wang Q, Xu L (2013). Targeted delivery of neurogenin-2 protein in the treatment for cerebral ischemia-reperfusion injury. Biomaterials.

[CR6] Dong B, Zhou B, Sun Z (2018). LncRNA-FENDRR mediates VEGFA to promote the apoptosis of brain microvascular endothelial cells via regulating miR-126 in mice with hypertensive intracerebral hemorrhage. Microcirculation.

[CR7] Duncan JA, Canna SW (2018). The NLRC4 inflammasome. Immunol Rev..

[CR8] Feigin VL (2005). Stroke epidemiology in the developing world. Lancet (London, England).

[CR9] Fink SL, Bergsbaken T, Cookson BT (2008). Anthrax lethal toxin and Salmonella elicit the common cell death pathway of caspase-1-dependent pyroptosis via distinct mechanisms. Proc Natl Acad Sci USA.

[CR10] Galligan JT, Martinez-Noël G, Arndt V, Hayes S, Chittenden TW, Harper JW, Howley PM (2015). Proteomic analysis and identification of cellular interactors of the giant ubiquitin ligase HERC2. J Proteome Res.

[CR11] Gross O, Thomas CJ, Guarda G, Tschopp J (2011). The inflammasome: an integrated view. Immunol Rev.

[CR12] Grote P, Wittler L, Hendrix D, Koch F, Waehrisch S, Beisaw A, Macura K, Blaess G, Kellis M, Werber M (2013). The tissue-specific IncRNA Fendrr Is an essential regulator of heart and body wall development in the mouse. Dev Cell.

[CR13] Guo Q, Wu Y, Hou Y, Liu Y, Liu T, Zhang H, Fan C, Guan H, Li Y, Shan Z (2018). Cytokine secretion and pyroptosis of thyroid follicular cells mediated by enhanced NLRP3, NLRP1, NLRC4, and AIM2 inflammasomes are associated with autoimmune thyroiditis. Front Immunol.

[CR14] Hong P, Li F-X, Gu R-N, Fang Y-Y, Lai L-Y, Wang Y-W, Tao T, Xu S-Y, You Z-J, Zhang H-F (2018). Inhibition of NLRP3 inflammasome ameliorates cerebral ischemia-reperfusion injury in diabetic mice. Neural Plast.

[CR15] Hu M, Ye P, Liao H, Chen M, Yang F (2016). Metformin protects H9C2 cardiomyocytes from high-glucose and hypoxia/reoxygenation injury via inhibition of reactive oxygen species generation and inflammatory responses: role of AMPK and JNK. J Diabetes Res..

[CR16] Huang L, Li G, Feng X, Wang L (2015). 15d-PGJ2 reduced microglia activation and alleviated neurological deficit of ischemic reperfusion in diabetic rat model. Biomed Res Int.

[CR17] Kun-Peng Z, Xiao-Long M, Chun-Lin Z (2017). LncRNA FENDRR sensitizes doxorubicin-resistance of osteosarcoma cells through down-regulating ABCB1 and ABCC1. Oncotarget.

[CR18] Li X, Wu Z, Fu X, Han W (2014). lncRNAs: Insights into their function and mechanics in underlying disorders. Mutat Res Rev Mutat Res.

[CR19] Ma Y, Wang J, Wang Y, Yang GY (2017). The biphasic function of microglia in ischemic stroke. Prog Neurobiol.

[CR20] Mao X, Sethi G, Zhang Z, Wang Q (2018). The emerging roles of the HERC ubiquitin ligases in cancer. Curr Pharm Des.

[CR21] Mizuma A, Yenari MA (2017). Anti-inflammatory targets for the treatment of reperfusion injury in stroke. Front Neurol.

[CR22] Poh L, Kang SW, Baik SH, Ng GYQ, She DT, Balaganapathy P, Dheen ST, Magnus T, Gelderblom M, Sobey CG (2019). Evidence that NLRC4 inflammasome mediates apoptotic and pyroptotic microglial death following ischemic stroke. Brain Behav Immun.

[CR23] Poyet JL, Srinivasula SM, Tnani M, Razmara M, Fernandes-Alnemri T, Alnemri ES (2001). Identification of Ipaf, a human caspase-1-activating protein related to Apaf-1. J Biol Chem.

[CR24] Qin X, Lu M, Zhou Y, Li G, Liu Z (2019). LncRNA FENDRR represses proliferation, migration and invasion through suppression of survivin in cholangiocarcinoma cells. Cell cycle (Georgetown, Tex).

[CR25] Sánchez-Tena S, Cubillos-Rojas M, Schneider T, Rosa JL (2016). Functional and pathological relevance of HERC family proteins: a decade later. Cell Mol Life Sci CMLS.

[CR26] Stegner D, Klaus V, Nieswandt B (2019). Platelets as modulators of cerebral ischemia/reperfusion injury. Front Immunol.

[CR27] Sun J, Wang F, Ling Z, Yu X, Chen W, Li H, Jin J, Pang M, Zhang H, Yu J (2016). Clostridium butyricum attenuates cerebral ischemia/reperfusion injury in diabetic mice via modulation of gut microbiota. Brain Res.

[CR28] Wang LQ, Zhou HJ (2018). LncRNA MALAT1 promotes high glucose-induced inflammatory response of microglial cells via provoking MyD88/IRAK1/TRAF6 signaling. Sci Rep.

[CR29] Wu R, Li X, Xu P, Huang L, Cheng J, Huang X, Jiang J, Wu LJ, Tang Y (2017). TREM2 protects against cerebral ischemia/reperfusion injury. Mol Brain.

[CR30] Wu H, Tang C, Tai LW, Yao W, Guo P, Hong J, Yang X, Li X, Jin Z, Ke J (2018). Biosci Rep.

[CR31] Xu TP, Huang MD, Xia R, Liu XX, Sun M, Yin L, Chen WM, Han L, Zhang EB, Kong R (2014). Decreased expression of the long non-coding RNA FENDRR is associated with poor prognosis in gastric cancer and FENDRR regulates gastric cancer cell metastasis by affecting fibronectin1 expression. J Hematol Oncol.

[CR32] Yao X, Yao R, Huang F, Yi J (2019). LncRNA SNHG12 as a potent autophagy inducer exerts neuroprotective effects against cerebral ischemia/reperfusion injury. Biochem Biophys Res Commun.

[CR33] Yin P, Wei Y, Wang X, Zhu M, Feng J (2018). Roles of specialized pro-resolving lipid mediators in cerebral ischemia reperfusion injury. Front Neurol.

[CR34] Yu X, Xu X, Jackson A, Sun J, Huang P, Mao Y, Chen Z, Lou M, Jiang Q, Zhang M (2016). Blood brain barrier disruption in diabetic stroke related to unfavorable outcome. Cerebrovasc Dis (Basel, Switzerland).

[CR35] Zhao Y, Shao F (2015). The NAIP-NLRC4 inflammasome in innate immune detection of bacterial flagellin and type III secretion apparatus. Immunol Rev.

[CR36] Zhao B, Yuan Q, Hou JB, Xia ZY (2019). Inhibition of HDAC3 ameliorates cerebral ischemia reperfusion injury in diabetic mice in vivo and in vitro. J Diabetes Res..

